# Design of GPU Network-on-Chip for Real-Time Video Super-Resolution Reconstruction

**DOI:** 10.3390/mi14051055

**Published:** 2023-05-16

**Authors:** Zhiyong Peng, Jiang Du, Yulong Qiao

**Affiliations:** 1 School of Optoelectronic Engineering, Guilin University of Electronic Technology, Guilin 541004, China; 21082304016@mails.guet.edu.cn; 2 School of Information and Communication Engineering, Harbin Engineering University, Harbin 150001, China; qiaoyulong@hrbeu.edu.cn

**Keywords:** super-resolution, deep learning, lookup table, GPU network-on-chip, real-time processing

## Abstract

Deep learning has a better output quality compared with traditional algorithms for video super-resolution (SR), but the network model needs large resources and has poor real-time performance. This paper focuses on solving the speed problem of SR; it achieves real-time SR by the collaborative design of a deep learning video SR algorithm and GPU parallel acceleration. An algorithm combining deep learning networks with a lookup table (LUT) is proposed for the video SR, which ensures both the SR effect and ease of GPU parallel acceleration. The computational efficiency of the GPU network-on-chip algorithm is improved to ensure real-time performance by three major GPU optimization strategies: storage access optimization, conditional branching function optimization, and threading optimization. Finally, the network-on-chip was implemented on a RTX 3090 GPU, and the validity of the algorithm was demonstrated through ablation experiments. In addition, SR performance is compared with existing classical algorithms based on standard datasets. The new algorithm was found to be more efficient than the SR-LUT algorithm. The average PSNR was 0.61 dB higher than the SR-LUT-V algorithm and 0.24 dB higher than the SR-LUT-S algorithm. At the same time, the speed of real video SR was tested. For a real video with a resolution of 540×540, the proposed GPU network-on-chip achieved a speed of 42 FPS. The new method is 9.1 times faster than the original SR-LUT-S fast method, which was directly imported into the GPU for processing.

## 1. Introduction

The demand of high-resolution (HR) images is growing for the advancement of information technology and modern industrial civilization, but image clarity is limited by the functionality of sensors and processors. The goal of super-resolution (SR) is to convert low-resolution (LR) images into high-resolution (HR) images, which has important research value. The deep learning SR method is a hot topic of research because it can achieve impressive results. In 2014, Dong et al. [1] first proposed the SRCNN method to achieve image SR reconstruction; many scholars then carried out further research based on SRCNN, such as FSRCNN [2], LapSRN [3], IMDN [4,5,6,7], etc. These methods achieved good results in the peak signal-to-noise ratio (PSNR), but usually cannot be implemented in real-time because of a large computational burden. Despite the availability of specialized computing engines that can enhance efficiency, the fixed “black box” network models make it is difficult to accelerate the calculation. The high hardware requirements and large computational costs hinder the wide application of deep learning SR algorithm.

In recent years, significant progress has been made in the hardware deployment of SR technology. Researchers have gradually applied SR technology to various hardware platforms by optimizing network structures, network quantization, and hardware acceleration methods. Lightweight network structures have notable advantages in hardware deployment. For example, Lim et al. [8] proposed that the EDSR, which is based on deep residual networks, has an improved and optimized network structure, providing a reference for hardware deployment. Hui et al. [4] proposed the IMDN, another lightweight and efficient SR model suitable for hardware deployment. However, these methods still face challenges in terms of inference speed when dealing with larger input images. One important reason is that the form of CNN convolution is a sliding window, and optimizing the network does not fundamentally improve the computational efficiency of the convolution. Cao et al. [9] proposed the matrix multiplication (MatMul) method and WinoConv [10] as alternatives to convolutional computations for SR hardware acceleration. They save the parameters of the convolution kernel in a LUT and then convert the input image into a vector for matrix operations to obtain the final output. This method is theoretically feasible; however, the process of converting images to vectors also consumes a significant amount of time. Moreover, if the MatMul method is to replace the sliding window effect of the convolution kernel applied to the image, the converted vector needs to contain a large amount of redundant data. Consequently, memory usage will increase exponentially as the image size grows.

With the widespread application of Graphics Processing Units (GPUs) in high-performance computing and artificial intelligence applications, designing efficient, low-power, and highly scalable on-chip communication networks is crucial. Modern GPUs have thousands of cores that require fast and scalable communication networks to support high-bandwidth, low-latency data exchange. Traditional on-chip buses and direct interconnect structures cannot meet these demands, and therefore, Network-on-Chip (NoC) technology has been widely applied in GPUs. NoC technology in GPUs faces a range of challenges, such as striking a balance between high performance and low power consumption, coping with uncertainty and faults, and meeting real-time and predictability requirements [11]. To address these challenges, researchers have proposed many novel approaches and techniques, such as heterogeneous NoCs, fault-tolerant techniques, and machine learning-based optimization strategies [12].

In image processing, the lookup tables (LUTs) have exhibited excellent computational performance and have been widely used for various color conversion tasks [13]. Storing the trained network parameters as an LUT provides more operability for optimizing the performance of post-algorithms. Deep learning methods based on LUTs have also emerged in low-level vision tasks [14,15,16,17]. In the image enhancement field, Zeng et al. [14] first proposed image-adaptive 3D lookup tables to achieve high-performance photo enhancement. On this basis, Wang et al. [15] considered spatial information and further proposed learnable spatial-aware 3D lookup tables. Wang et al. [16] model local context cues and propose pixel-adaptive lookup table weights for portrait photo retouching. Recently, some scholars have applied the LUT to the image restoration field [17], but the redundant calculations on the CPU still hinder the speed. Due to the strong operability of the LUT, this paper combines the working principle of the LUT with features of GPU to process image data. The primary focus of this research involves the design of deep learning algorithms combined with LUT and the parallel implementation of GPU network-on-chip.

In summary, the contributions of this paper are:

(1) The paper proposes a deep learning SR algorithm combined with LUT, which enables the algorithm to be efficiently implemented on the GPU.
(2) The proposed video SR algorithm has been implemented in real-time on a GPU by elaborate optimization strategy: storage access optimization, conditional branching function optimization, and threading optimization.

The remaining paper is organized as follows. Section 2 designs a SR network based on deep learning and LUT. Section 3 discusses the implementation of a parallel SR algorithm on GPU by optimization. Section 4 is about the algorithm testing and result analysis. Section 5 summarizes the major findings and concludes of the paper.

## 2. Design of a SR Network Based on Deep Learning and LUT

### 2.1. Design of Video SR Network

**Figure 1:**
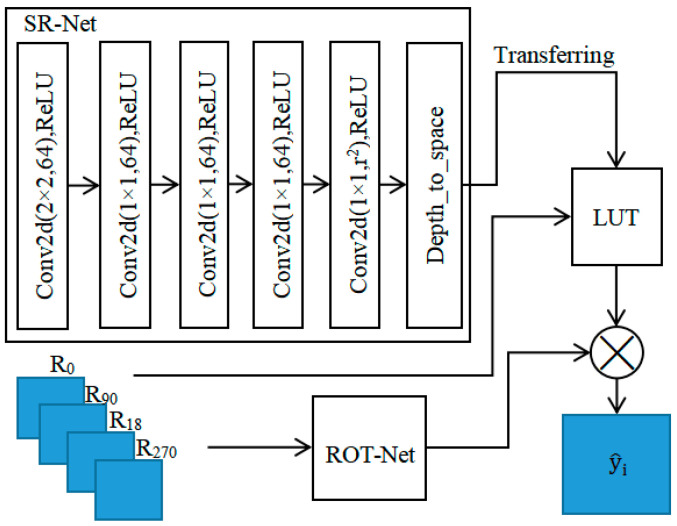
Structure of the SR network.

The proposed SR network structure is shown in Figure 1 by combining deep learning and LUT. The new SR network is composed of SR-Net [17] and ROT-Net. SR-Net is a deep learning SR network with a receptive field (RF) size of 4. The SR-Net model is first trained, and then its output is passed to the LUT. ROT-Net is a feature extraction network, which is used to get the weight information at different rotation angles. SR-Net primarily learns the feature information from LR images to HR images, while ROT-Net mainly learns the pixel weight information for weighted fusion of SR results at different angles.

There is an exponential increase in the size of the LUT with the RF size. In practice, if the RF size is 3 or greater, the complete LUT can become very large. To mitigate the size of the LUT, a uniform sampling method can be employed to reduce the RF size. In the paper, a lightweight neural network SR-Net with an RF size of 4 is designed. SR-Net consists of a convolutional neural network (CNN) with 6 convolutional layers, in which the first layer has a kernel size of 2 × 2 and the rest have a kernel size of 1 × 1. The number of output channels for the first 5 convolutional layers is set to 64, and the number of output channels for the last layer is set to the square of the image upscaling factor.

To enhance the extraction of local structural features at different angles and increase the RF size, this paper adopts the method proposed by Jo et al. [17]. The image is rotated to 0, 90, 180, and 270 degrees, and the four outputs of the rotated images are fused to obtain the SR result. The rotational strategy has been used to maximize the accuracy only at the test time in previous deep SR works [8,18]. In the original fusion method, the average operation is used according to the Formula (1). Formally, the final output, ${\hat{y}}_{i}$, can be expressed as follows:

(1) $${\hat{y}}_{i}=\sum_{j=0}^{3}{A_{j}(R}_{j}^{-1}(f(R_{j}(x_{i})))),$$

in which ${\hat{y}}_{i}$ is the final output of SR reconstruction, $x_{i}$ is LR input patch, $A_{j}$ is the coefficients for different angles, f is the deep SR network, $R_{j}$ is image rotation operation to j×90 degree, and $R_{j}^{-1}$ is the reverse rotation operation.

**Figure 2:**
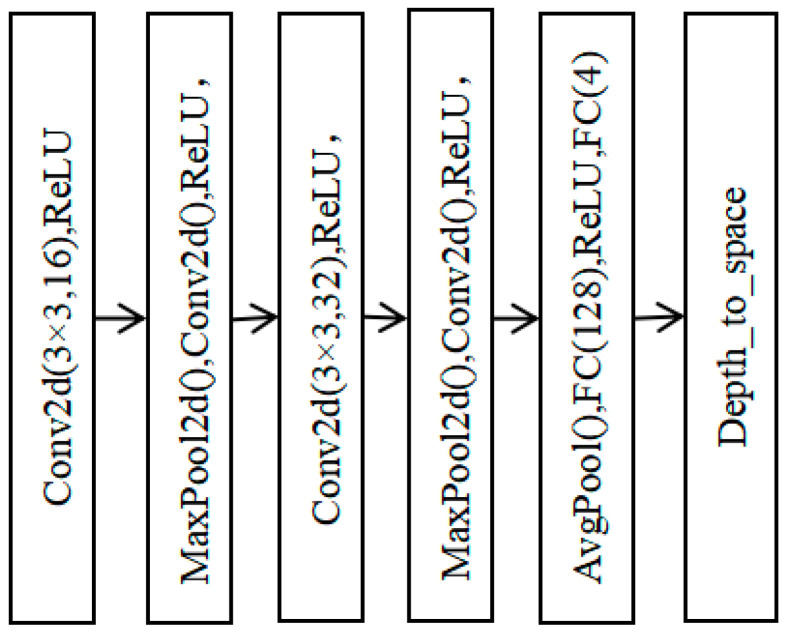
Structure of the ROT-Net network.

In the paper, considering that the local texture structure used in SR has directionality, the weights at different directions should be different. Therefore, the coefficient value, $A_{j}$. for each pixel at different angles is obtained through a lightweight CNN called ROT-Net, whose network structure is shown in Figure 2. ROT-Net consists of three convolutional layers and two fully connected layers, in which the ReLU activation function and max pooling operation are used for feature extraction. To adapt to images with different scales, an adaptive average pooling is introduced to fix the feature size. Finally, the four results from the LUT are fused according to the weights from ROT-Net, and a SR result with more detailed information is obtained.

The training process of the network comprises two stages. In the first stage, a lightweight SR-Net network is trained using the DIV2K dataset to obtain complete LUT parameters. In the second stage, we fixed the parameters of the SR-Net and trained the ROT-Net. The four results with different rotation angles from the LUT were fused according to the adaptive weights from ROT-Net to generate the final SR image.

### 2.2. LUT and Interpolation Design

**Figure 3:**
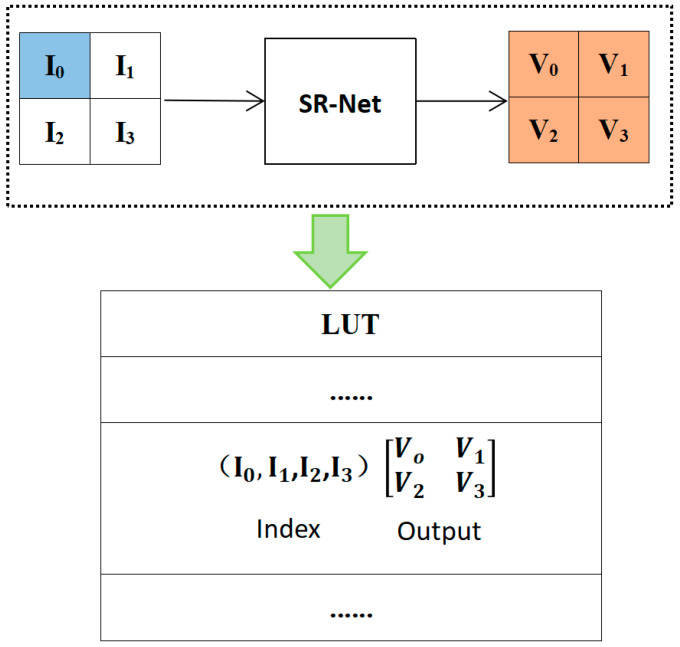
The mapping method of the LUT from SR-Net.

After training the SR-Net network, the LUT is designed for a SR-Net network with an RF size of 4. The mapping method of the LUT is shown in Figure 3. The output values of the network are indexed by four corresponding input values and saved in the LUT. As illustrated in Figure 3, the LUT is the term 4D-LUT, which consists of four input values and two output values.

Once the LUT is built, SR is performed solely with the LUT. However, mapping the complete network parameters to the LUT will consume a significant amount of storage resources. If the dimensions of the LUT increase, the storage resource requirements increase exponentially. By uniformly sampling for the original input space of $2^{8}$ bins (0–255 for an 8-bit input image), the size of the LUT can be reduced. For example, a 4D-LUT can be divided into $2^{4}+1$ bins as uniformly sampled spaces by using an interval of $2^{4}$. The overall size will be reduced from 64 G (4D-LUT[256][256][256][256]) for the complete LUT to 1.274 MB (4D-LUT[17][17][17][17]). After uniform sampling, the entire LUT process is divided into two specific steps: calculating the original quantized coordinates and performing interpolation operations. The interpolation coordinates are calculated from the original quantized coordinates and weights.

**Figure 4:**
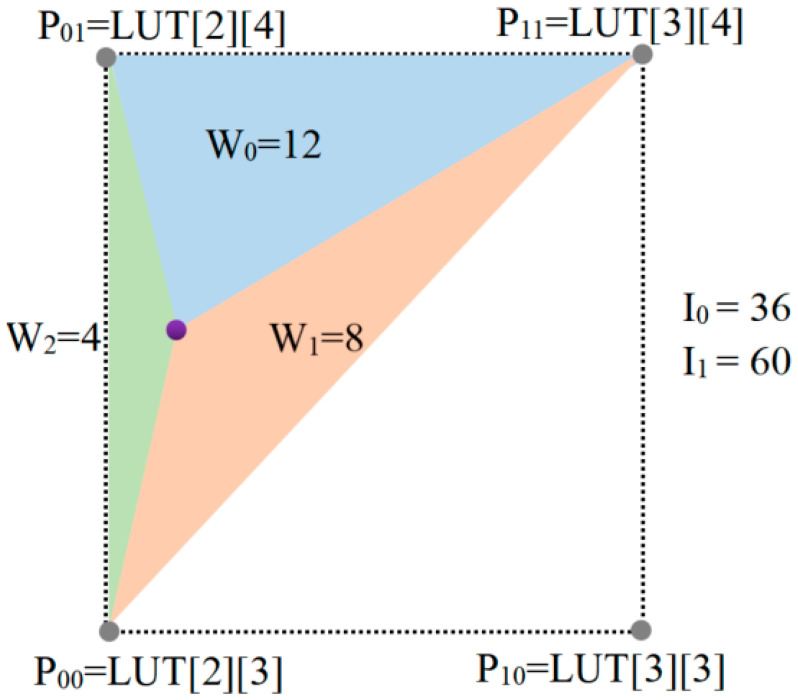
An example of triangular interpolation for a sampled 2D-LUT with a sampling interval of $2^{4}$.

After obtaining the original quantized coordinates, an interpolation calculation is performed using the tetrahedral interpolation method [19]. A tetrahedron is formed by four non-coplanar three-dimensional coordinate points, and interpolation is performed on the four triangles formed by the tetrahedron. Based on the weight of the interpolation point relative to the four vertices of the tetrahedron, the final interpolation result is achieved by weighting the interpolation results of individual triangles. An example of triangular interpolation is a sampled 2D-LUT with the sampling interval of $2^{4}$. An example of interpolation for a sampled 2D-LUT with the sampling interval of 4 is shown in Figure 4. For the query input, $I_{0}=24$ and $I_{1}=60$, the nearest points, $P_{00}$, $P_{01}$, and $P_{11}$, and the corresponding weights, $w_{0}$, $w_{1}$, and $w_{2}$, are determined. The output value is calculated as the weighted sum. The same principle applies to 3D and 4D LUTs.

In Figure 4 (the 2D equivalent triangular interpolation), first, the input values $I_{0}=36{(00100100}_{\left(2\right)})$ and $I_{1}=60(00111100_{\left(2\right)})$ are split into the 4 most significant bits (MSBs) and 4 least significant bits (LSBs). The MSBs of $I_{0}$ and $I_{1}$ are 2 and 3, respectively, which are used to determine the nearest sampling point. The LSBs of $I_{0}$ and $I_{1}$ are $L_{x}=4$ and $L_{y}=12$, respectively, which are used to determine the weights of the boundary triangle and boundary vertices. Two boundary vertices are fixed at $P_{00}=\mathrm{LUT}\left[2\right][3]$ and $P_{11}=\mathrm{LUT}\left[2+1\right][3+1]$, and the third vertex is determined by comparing $L_{x}$ and $L_{y}$. Here, we choose $P_{01}=\mathrm{LUT}\left[2\right][3+1]$. The weight of each vertex is proportional to the area of the corresponding triangle, which can be calculated as: $w_{0}=2^{4}-L_{y}$, $w_{1}=L_{y}-L_{x}$, and $w_{2}=L_{x}$. The predicted output value of this 2D-LUT example is: $\hat{V}=(w_{0}P_{00}+w_{1}P_{01}+w_{2}P_{02})/2^{4}$.

The same principle applies to 4D LUTs. Based on the LSB values (corresponding to $L_{x}$, $L_{y}$, $L_{z}$, and $L_{t}$ for $I_{o}$, $I_{1}$, $I_{2}$, and $I_{3}$, respectively), out of the 24 possible combinations of $L_{x}$, $L_{y}$, $L_{z}$, and $L_{t}$, one branch is selected as the predicted output value using a judgement mechanism. Based on the coordinate information and using tetrahedral interpolation, the predicted output value $\hat{V}$ of the 4D-LUT is calculated using the Formula (2).

(2) $$\hat{V}=\frac{1}{W}\sum_{i=0}^{4}w_{i}P_{i},$$

where, W is the sampling interval, $w_{i}$ is the weight assigned to the interpolated point with respect to sampling coordinate points, and $P_{i}$ is the set of all interpolation coordinate points.

As the input LR image size increases, the computation time for SR using LUTs grows exponentially. Therefore, optimizing the LUT structure and interpolation algorithm is extremely important.

## 3. GPU On-Chip Optimization Implementation of the Algorithm

GPU is a high-performance computing device. In 2007, NVIDIA introduced the Compute Unified Device Architecture (CUDA). To deploy the algorithm on a GPU and implement it using a GPU NoC, we adopted the CUDA programming framework, which supports GPU computing. Various storage systems have been designed in CUDA [20,21,22], including global memory, shared memory, and constant memory, among others. The storage systems are used at different locations in the algorithm, based on their storage capacity and access speed. The effective allocation of data storage space plays a crucial role in enhancing the overall performance of the algorithm. In this study, the quantized coordinate information of each pixel and the final interpolation process are obtained through a LUT, which requires traversing all pixels in the image. If executed in serial, its performance cannot meet the real-time requirements. Therefore, the computation of original quantized coordinates and the final SR process are realized by GPU parallel. In addition, three optimization strategies are used to optimize the GPU parallel algorithm for video SR scenarios.

### 3.1. Implementation of the Parallel SR Algorithm Based on CUDA

In the new SR algorithm, based on the LUT, the tetrahedral interpolation method is used according to the original quantized coordinates and weights. The interpolation coordinates can be directly obtained for sampling points, but the nearest neighbor points need to be calculated for non-sampling points. For example, the original quantized coordinates P (the neighbor points) include: in 2D, only $P_{00}$, $P_{01}$, $P_{10}$, and $P_{11}$; in 3D, 8 neighboring points from $P_{000}$ to $P_{111}$; and in 4D, 16 neighboring points from $P_{0000}$ to $P_{1111}$. Thus, if the dimension of the LUT increases, the number of calculated neighboring points will also increase. In the paper, two kernel functions, PCoordinatesKernel and SRLinearKernel, are designed. The PCoordinatesKernel obtains the coordinates P by performing a lookup in the LUT using the input image data. The SRLinearKernel performs image SR by interpolating the original image using coordinate data.

For 4D-LUT, in order to facilitate data manipulation in CUDA, image data is compressed into a one-dimensional matrix. A one-dimensional coordinate matrix $p_{i}$ is obtained by querying a LUT, which can be represented as Formula (3):

(3) $$p_{i}=\mathrm{LUT}\left(b\right)\times r^{2}+i,$$

where, b is the set of index values corresponding to the higher four bits of the image pixel space on the LUT, r represents the magnification factor, and i corresponds to the number of pixels after a single pixel SR (equal to all integers between 0 and $r^{2}$).

**Figure 5:**
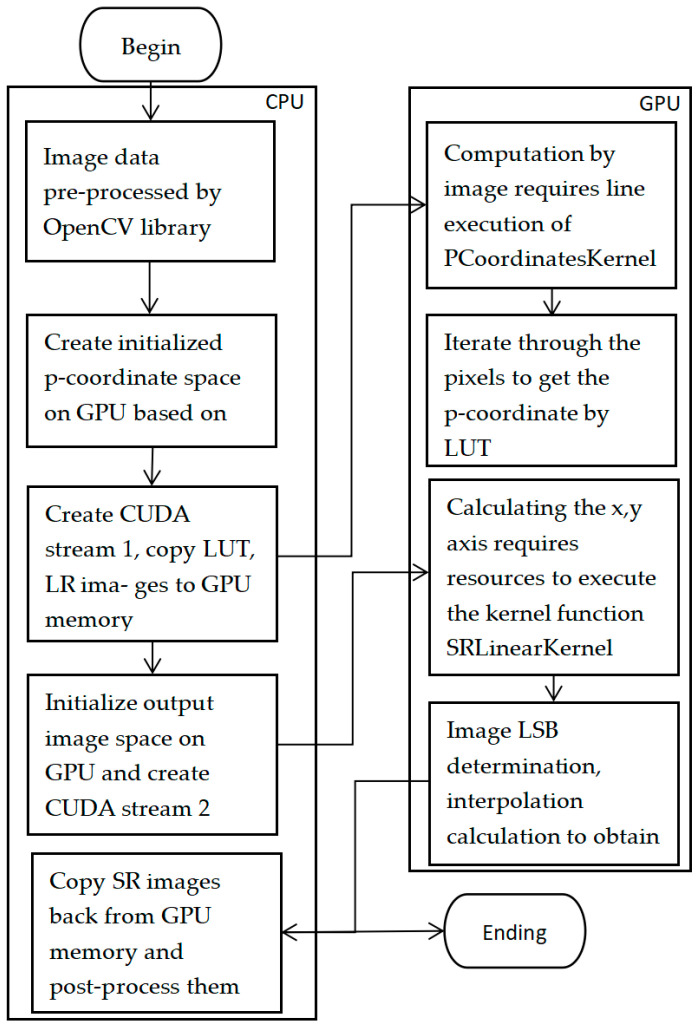
Flow chart of optimized CPU/GPU LUT for SR.

The final original quantized coordinates P are represented as follows:

(4) $$P=\left\{p_{i}\_o|0<o<2^{R}\right\},$$

where o is the number of coordinates and R is the dimension of the LUT. The coordinates of the current pixel are fixed by the index value in the CUDA thread. We set block_size = k (0 < k ≤ 1024) and grid_size = (total number of pixels + k − 1)/k; block_size is the thread block size of a single grid, and grid_size is the grid size allocated by the kernel function. Using the built-in one-dimensional indexing variables blockDim.x, blockIdx.x, and *threadIdx.x* in CUDA, image pixel traversal can be accomplished. blockDim.x represents the number of threads contained in a thread block. blockIdx.x is the index of the thread block in the grid, indicating the encoding of the current thread in its thread block. threadIdx.x is the index of the thread in its thread block [23]. The precise scheduling of GPU threads can be achieved by combining these three built-in variables. The thread index tidx/tidy can be calculated as Formula (5):

(5) $$\begin{array}{l} tidx=blockDim.x\times blockx.x+threadIdx.x,\\ tidy=blockDim.y\times blockx.y+threadIdx.y, \end{array}$$

where tidx and tidy denote the indexes mapped to the *x* and *y* directions of the thread block, respectively. The pre-processed image data and LUT data are inputted into the PCoordinatesKernel GPU kernel function to compute the coordinates, P, which are then used for the subsequent calculation of interpolated coordinates. The flow chart for SR of LUTs on the CPU/GPU is shown in Figure 5.

The interpolation process requires compressing the three-channel RGB image into a one-dimensional array and sending it to the SRLinearKernel kernel function. Its calculation process requires mapping from a three-dimensional matrix (Formula (2)) to a one-dimensional matrix. Specifically, the mapping to a one-dimensional coordinate space needs to be calculated for each of the RGB channels, followed by interpolation. The final predicted output of the compressed image data in one dimension is obtained in CUDA as follows:

(6) $$\begin{array}{l} R:{\hat{V}}_{r}(index_{r}+a)=w\times p[x\times w\times r^{2}+y\times r^{2}+H],\\ G:{\hat{V}}_{g}(index_{r}+a+index_{g})=w\times p[M+x\times w\times r^{2}+y\times r^{2}+H],\\ B:{\hat{V}}_{b}(index_{r}+a+index_{b})=w\times p[2\times M+x\times w\times r^{2}+y\times r^{2}+H], \end{array}$$

where, R, G, and B represent the interpolation of three channels and the ${\hat{V}}_{r,g,b}$ is the one-dimensional data of the final output image. $index_{r}=\left(x\times h+y\right)\times 4^{2}$, x and y correspond to the coordinates on the x-axis and y-axis, respectively, and h and w represent the width and height of the image. $M=h\times w\times r^{2}$, the size of a channel image. H represents the two-dimensional coordinate matrix $H\left[i,j\right]=i\times r+j(i,j\;between\;0-(r-1))$ where the interpolated values are located. The specific coordinate, P, chosen needs to be calculated by comparing the values of the LSB.

In practical applications, when traversing an input image, the current pixel position needs to be determined through a two-dimensional coordinate. Therefore, it is necessary to map the two-dimensional coordinates to the index of GPU threads. According to Equation (5), we use tidx and tidy to denote the index of image pixels mapped to threads, respectively. For GPUs based on the Turing architecture, there are restrictions on the grid size and thread block size. The maximum values allowed for the grid size in the *x*, *y*, and *z* directions are $2^{31}-1$, 65,535, and 65,535, respectively. The maximum values allowed for the thread block size in the *x*, *y*, and *z* directions are 1024, 1024, and 64, respectively, and the product of the sizes in the three directions cannot exceed 1024. Regardless of how it is defined, a thread block can have a maximum of 1024 threads [24].

### 3.2. Optimization Strategy for Algorithms on CUDA

The dimension of the LUT is higher, the number of calculated coordinates and the number of conditional branches in the interpolation calculation will increase, requiring more resources and computing power. Optimizing the GPU parallel strategy is crucial. It is important to minimize the data transfer between CPU and GPU, to increase the arithmetic strength of the kernel functions, and to increase the parallel scale of the kernel functions [24]. In this paper, three specific optimization strategies are used to achieve efficient data transfer, enhance the arithmetic strength of the kernel function, and increase the kernel function parallelism scale of the algorithm, as follows:

(1) Storage access optimization

During the computation of the original quantized coordinates, it is necessary to allocate the memory for the LUT, image data, and the required quantization coordinates on the GPU simultaneously. In the case of video SR, SR for each frame of the video should not create and destroy all data memory as often, as some of the data memory can be applied to the calculation of SR for all frames. For example, by copying the LUT to the GPU only once, with all frames accessing that LUT, the SR of each frame will save time by copying the LUT to the GPU once. Global memory in GPUs has a large capacity, typically greater than 2 GB and sometimes even larger, and is accessible to all threads. However, because transferring data between CPU and GPU takes a significant amount of time, it is necessary to organize data transfer in memory based on the characteristics of the data.

Once the image size is known in a video stream, the original quantized coordinates, P, and the size of the output result can be calculated. The storage access optimization adopted in this paper mainly includes the following:

In the CUDA kernel function, repeated memory allocation, and resource recovery are reduced by modifying data in memory. The memories for LUT, quantization coordinates, P, and the final output result are accordingly allocated on the GPU, and their storage size is fixed and calculated based on the input image. These memories are not released before thread exit, thus saving time for memory initialization and data transfer between the host and device.

Accessing global memory in CUDA is comparatively slow, and computing coordinates and interpolation requires multiple accesses to image data and interpolation coordinate data stored in GPU memory. To solve this problem, the built-in function __ldg() in CUDA can be used to read data from global memory, and by specifying read-only caching with __ldg(), the GPU can directly access the data from the faster Texture Cache. Using function __ldg() in CUDA can reduce latency and improving the efficiency of global memory data access.

(2) Conditional branching function optimization

In the interpolation process, the interpolation coordinates need to judge the LSB in different pixel spaces to determine the weights of the boundaries and boundary vertices. However, the frequently used if branching statements in the code make different threads execute different instruction paths, which will result in more time and resources needed for thread synchronization and cooperation. This paper improves the execution efficiency of the CUDA program by using high-speed bitwise and logical operations instead of branching statements. For example, if the LSBs of the image are calculated as fa and fb, their difference can be calculated and right-shifted 31 bits. If fa is greater than fb, the highest bit of the difference is 0; otherwise, it is 1. The logical AND operator is applied to the operation that needs to be executed. If the flag is 1, the operation is executed; otherwise, it is not.

(3) Threading optimization

**Figure 6:**
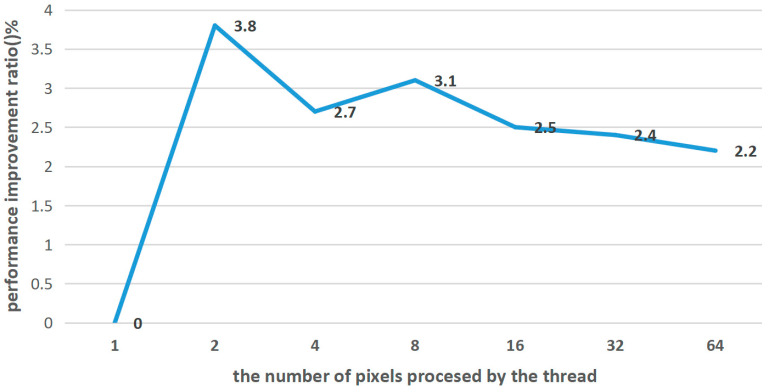
The performance improvement ratio when the number of pixels processed by threads varies.

**Figure 7:**
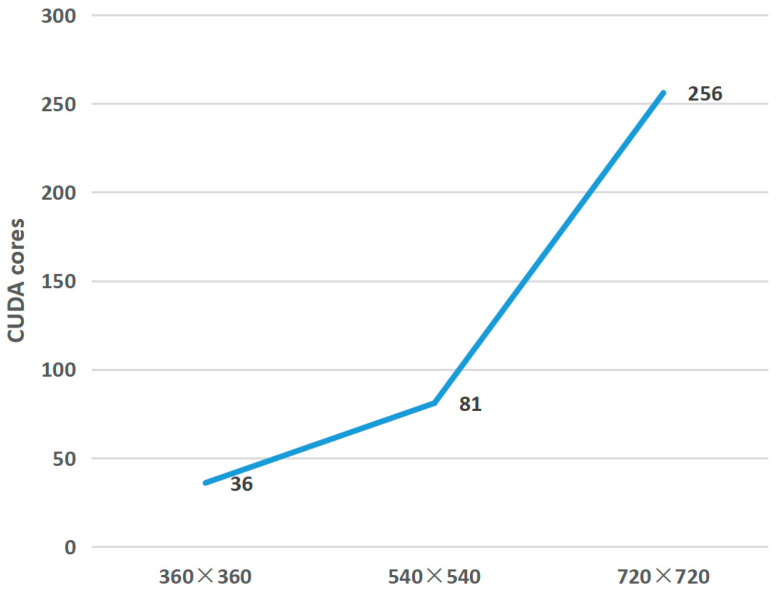
Minimum number of CUDA cores required for different resolutions.

In RTX 3090GPU, each Streaming Multiprocessor (SM) can support up to 2048 thread blocks and each thread block can support up to 1024 threads. However, the number of thread blocks is also limited by the amount of shared memory and registers available. If a fixed number of thread-blocks and grid sizes are used, it may lead to wasted resources or an insufficient thread scale. The thread-dispatch experiment in Figure 6 of the paper tested the relationship between the number of threads and pixels in the GPU. Therefore, an adaptive calculation should be performed when allocating the number of threads in CUDA. The adaptive calculation formula is as Formula (7):

(7) $$\begin{array}{l} G_{x}=(w+blockIdx.x-1)/blockIdx.x,\\ G_{y}=(h+blockIdx.y\times n-1)/(blockIdx.y\times n)\times n/2, \end{array}$$

where, $G_{x}$ and $G_{y}$ represent the corresponding grid sizes in the x and y directions, respectively, and n represents the number of pixels processed by a single thread. The maximum thread block size is 1024 (32×32). For the stability of the program and to avoid resource waste, a grid size of ($G_{x}$, $G_{y}$) and thread block size of (32, 32) are allocated adaptively within constraints. This adaptive allocation can achieve good performance for any image size. However, if on low-performance hardware, reducing the grid size and thread block size is necessary to lower the number of CUDA cores and achieve real-time performance. Figure 7 shows the minimum number of CUDA cores required to reach a frame rate of over 20 FPS for various image sizes.

Since the LUT SR process requires rotating the image four times (0, 90, 180, and 270 degrees), calling the kernel function four times for image interpolation would take a considerable amount of time. Using CUDA Stream to asynchronously execute kernel functions allows multiple kernel functions to be executed simultaneously. By using the CUDA Stream asynchronous mechanism, device waiting time for the host can be reduced, thereby improving overall performance.

## 4. Experimental Results and Analysis

In this study, the CPU used in the experimental environment is Intel(R) Core (TM) i9-10900k, and the GPU used is RTX 3090. The experimental platform is Ubuntu18.04. The experimental includes algorithm ablation experiments, comparison tests with classical SR algorithms, and real video image testing.

### 4.1. Algorithm Ablation Experiment

#### 4.1.1. Thread Allocation Ablation Experiment

The test time for obtaining the output result of SR using LUT before optimization with an input image resolution of 540 × 540 is 22.8 ms, and the resolution of the output image is 2160 × 2160 (×4). We set the number of threads in a block to 1024 and used the thread multi-element revisit strategy [25] to allow GPU threads to process multiple pixels. Figure 6 shows the performance improvement ratio when the number of pixels processed by threads varies. The performance improvement ratio L is calculated as Formula (8):

(8) $$L=\frac{\left(T_{bef}-T_{aft}\right)}{T_{aft}}\times 100\%,$$

where, $T_{bef}$ and $T_{aft}$ represent the execution time of a certain optimization before and after its effectiveness. From the Figure 6, it can be found that the method achieves a performance improvement ratio of 3.8% when a single GPU thread processes 2 pixels. This experiment proves that a single thread can handle multiple pixels, reducing the grid size. In the experiment, the overall GPU memory consumption is 2622 MiB (a memory usage rate is 10.7%). The results show that the new method can be implemented on lower-memory embedded hardware.

This paper achieves high GPU acceleration performance by using a small grid size. A smaller grid size means a reduced GPU load and scheduling, thereby improving the parallel computing efficiency of the algorithm. Moreover, a smaller grid size indicates good scalability of the algorithm, making it easier to implement on GPUs with lower computing power.

#### 4.1.2. Image Ablation Experiment with Different Resolution

We selected commonly seen low-resolution images in real-world scenarios, with pixel counts of 100,000, 300,000, 500,000, and 1,000,000, which correspond to resolutions ranging from 360 × 360 to 1080 × 1080, to test the performance. As the resolution increases, the number of computing units executed by the kernel also increases. The minimum required number of CUDA cores for the algorithm to run in real-time with different video resolutions is shown in Figure 7. The 360×360 resolution requires a minimum of 36 cores and achieves 39 FPS; the 540 × 540 resolution requires 81 cores and achieves 25 FPS; and the 720×720 resolution requires 256 cores and achieves 20 FPS.

**Figure 8:**
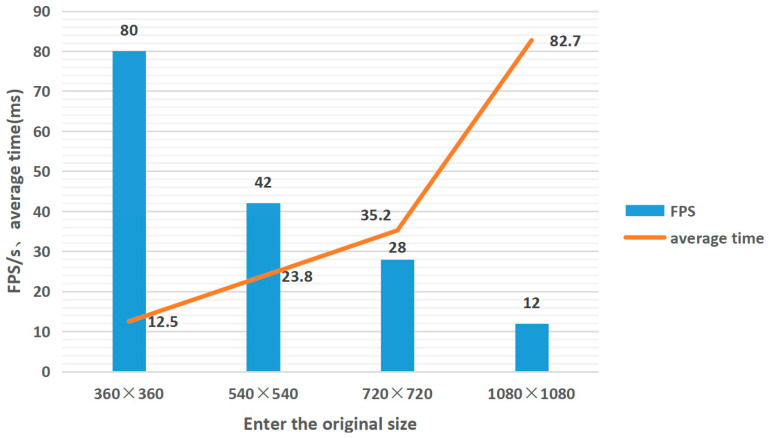
Real time SR frame rate and average timing.

As the input image size increases, the algorithm’s running time also increases. The speed tests are performed on images with different resolutions, and the test results are shown in Figure 8. The figure displays the consumed time (in milliseconds) and FPS for images of different resolutions. The bar chart represents the average FPS of the output displayed after the input image is SR four times, and the line chart represents the stable single-frame time consumption. The maximum frame rate was 80 FPS for the 360 × 360 image, and the average elapsed time was 12.5 ms per frame.

**Figure 9:**
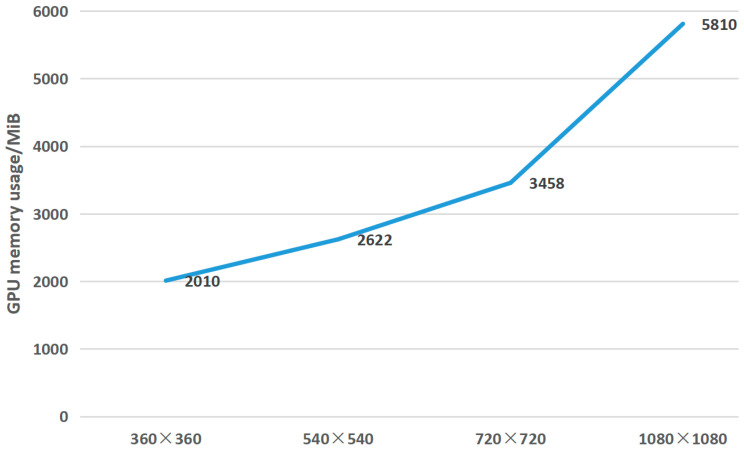
GPU memory usage of video streams with different resolutions.

As the resolution increases, the GPU memory usage also increases. The test results about GPU memory usage of video streams with different resolutions are shown in Figure 9. Among them, the minimum memory usage is 2010 MiB at a resolution of 360 × 360. With the increase of resolution, the memory usage shows a clear upward trend, and reaches 5810 MiB at a resolution of 1080×1080.

### 4.2. Network-on-Chip Performance Comparison with Classical SR Algorithms

Four public datasets that have been widely used in SR task evaluation, Set5, Set14, BSDS100 [26], and Urban100 [27], are used to compare the paper with classical algorithms. For quantitative evaluation, PSNR and structural similarity index (SSIM) [28] are used, which are traditionally used for image quality assessment. The paper compared our method with several classic SR algorithms. The runtime test was conducted by using a 320×180 LR image as input to generate a 1280×720 HR RGB image, and the average was obtained from 10 tests. Due to our successful optimization and deployment of the algorithm on the GPU, the execution environment is set to GPU, while other programs such as callers, data transmission, etc., are run on the CPU.

In Table 1, our proposed algorithm shows good PSNR and SSIM values on four datasets, which are higher than those of SR-LUT-S. The largest improvement in PSNR is achieved on the Set14 dataset, with an increase of +0.51 dB. At the same time, our algorithm runs 9.1 times faster than the SR-LUT-S fast algorithm, and even faster than the SR-LUT-V algorithm, with a stable output of 10 ms. We also employ GPUs for inference on other neural network models. Our algorithm is faster than mainstream lightweight neural networks. It is 5.5 times faster than the quickest lightweight neural network, IMDN, greatly surpassing the inference speed of lightweight neural networks.

**Figure 10:**
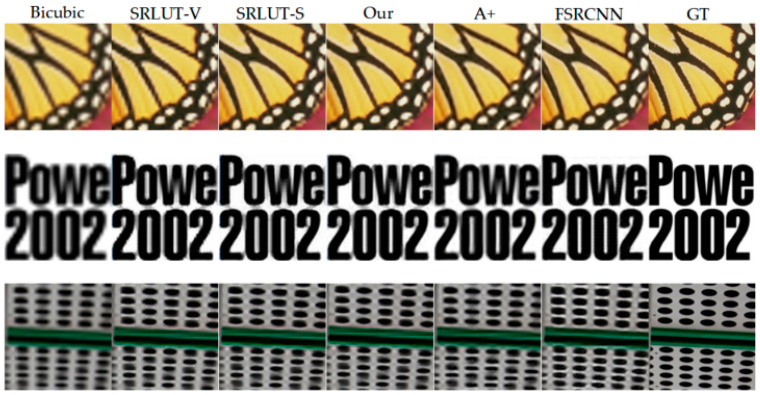
SR reconstruction by different methods.

The SR comparison results are shown in Figure 10. Based on visual inspection, the proposed method exhibits prominent restoration effects compared to bicubic interpolation and shows better sharpness in some detail recoveries than SR-LUT-V/S.

### 4.3. Real Video Testing

**Figure 11:**
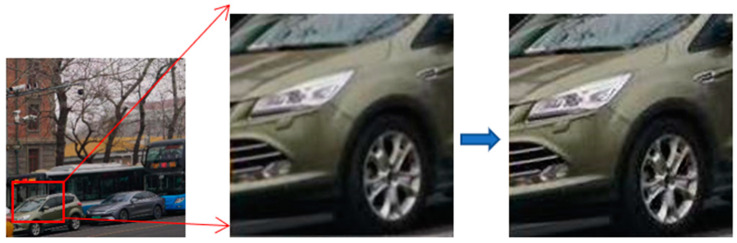

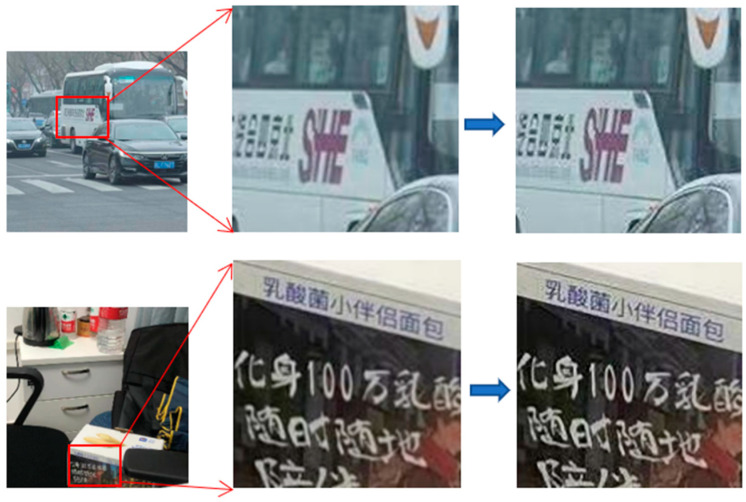
SR effect of real video.

In practical applications, this paper uses a ZWO ASI462 industrial camera to capture images in real-time. By sampling a 540 × 540 video area for SR, the algorithm generates a 2160 × 2160 result for display, achieving 42 FPS with a memory consumption of 2622 MiB. The representative results are shown in Figure 11. From the results, it can be seen that the reconstruction of SR using the method proposed in this paper can significantly improve the effects on lines and contours, and achieve completely real-time video SR reconstruction.

## 5. Conclusions

This paper explored the application of a LUT combined with deep learning for video SR, and optimized the SR algorithm through GPU implementation. Firstly, the design and training strategy of the SR network were described, which effectively utilized the influence of different rotation angles on the LUT. The weights of features from different angles are adaptively changed to improve the quality of the SR reconstructed image. The network parameters are uniformly sampled and added to a carefully designed multidimensional LUT. The video real-time SR reconstruction is achieved by fully utilizing the data characteristics of the LUT and the CUDA kernel function to execute a large number of threads in parallel. GPU optimization strategies are proposed, which include storage access optimization, conditional branching function optimization, and threading optimization. Finally, the test results show that the speed of the new SR is 9.1 times faster than SR-LUT-S. In a quantitative comparison on public datasets, the new method outperforms SR-LUT in terms of PSNR and SSIM. Additionally, the GPU-accelerated implementation of the new algorithm only requires 81 CUDA cores and 2622 MiB of GPU memory. Real-time ×4 SR of 540 × 540 resolution video streams can be achieved with 42 FPS. Theoretically, our method can also be applied to embedded hardware environments with lower computing power.

## Figures and Tables

**Table 1:** Comparison of test data results.

	Method	Runtime	Size	Set5		Set14		BSDS100		Urban100	
				PSNR	SSIM	PSNR	SSIM	PSNR	SSIM	PSNR	SSIM
Interpolation	Nearest	4 ms *	-	26.25	0.7372	24.65	0.6529	25.03	0.6293	22.17	0.6154
	Bilinear	16 ms *	-	27.55	0.7884	25.42	0.6792	25.54	0.6460	22.69	0.6346
	Bicubic	60 ms *	-	28.42	0.8101	26.00	0.7023	25.96	0.6672	23.14	0.6574
LUT	SR-LUT-V [17]	15 ms *	1 MB	29.22	0.8304	26.65	0.7258	26.33	0.6880	23.68	0.6852
	SR-LUT-F [17]	34 ms *	77 kB	29.77	0.8429	26.99	0.7372	26.57	0.6990	23.94	0.6971
	SR-LUT-S [17]	91 ms *	1.274 MB	29.82	0.8478	27.01	0.7355	26.53	0.6953	24.02	0.6990
	Our	10 ms ”	2.974 MB	29.89	0.8494	27.52	0.7614	26.89	0.7118	24.03	0.7082
Sparse coding	NE+LLE [29]	7016 ms *	1.434 MB	29.62	0.8404	26.82	0.7346	26.49	0.6970	23.84	0.6942
	Zeyde et al. [30]	8797 ms *	1.434 MB	26.69	0.8429	26.90	0.7354	26.53	0.6968	23.90	0.6962
	ANR [31]	1715 ms *	1.434 MB	29.70	0.8422	26.86	0.7368	26.52	0.6992	23.89	0.6964
	A+ [32]	1748 ms *	15.171 MB	30.27	0.8602	27.30	0.7498	26.73	0.7088	24.33	0.7189
DNN	FSRCNN [2]	75 ms ”	12 K ^†^	30.71	0.8656	27.60	0.7543	26.96	0.7129	24.61	0.7263
	CARN-M [33]	270 ms ”	412 K ^†^	31.82	0.8898	28.29	0.7747	27.42	0.7305	25.62	0.7694
	RRDB [6]	1780 ms ”	16,698 K ^†^	32.68	0.8999	28.88	0.7891	27.82	0.7444	27.02	0.8146
	EDSR [8]	130 ms ”	1300 K ^†^	32.46	0.8968	28.80	0.7876	27.71	0.7420	26.64	0.8033
	IMDN [4]	55 ms ”	715 K ^†^	32.21	0.8948	28.58	0.7811	27.56	0.7353	26.04	0.7838

*: denotes the running time tested on the CPU. ”: denotes the running time tested on the GPU. ^†^: denotes the number of deep neural networks (DNN) parameters.

## Data Availability

The public datasets we use are available from the web. Set5: https://paperswithcode.com/dataset/set5; Set14: https://paperswithcode.com/dataset/set14; BSDS100: https://paperswithcode.com/dataset/bsd; Urban100: https://paperswithcode.com/dataset/urban100 (all sites accessed on 8 April 2023).

## References

[1] Dong C., Loy C.C., He K., Tang X. Learning a Deep Convolutional Network for Image Super-Resolution. Proceedings of the European Conference on Computer Vision.

[2] Dong C., Loy C.C., Tang X. Accelerating the Super-Resolution Convolutional Neural Network. Proceedings of the European Conference on Computer Vision.

[3] Lai W., Huang J., Ahuja N., Yang M. Deep Laplacian Pyramid Networks for Fast and Accurate Super-Resolution. Proceedings of the IEEE Conference on Computer Vision and Pattern Recognition.

[4] Hui Z., Gao X., Yang Y., Wang X. Lightweight Image Super-Resolution with Information Multi-distillation Network. Proceedings of the 27th ACM International Conference on Multimedia.

[5] Li Z., Yang J., Liu Z., Yang X., Jeon G., Wu W. Feedback Network for Image Super-Resolution. Proceedings of the IEEE/CVF Conference on Computer Vision and Pattern Recognition.

[6] Wang X., Yu K., Wu S., Gu J., Liu Y., Dong C., Qiao Y., Loy C.C. ESRGAN: Enhanced Super-Resolution Generative Adversarial Networks. Proceedings of the European Conference on Computer Vision Workshops.

[7] Mei Y., Fan Y., Zhou Y., Huang L., Huang T.S., Shi H. Image Super-Resolution with Cross-Scale Non-Local Attention and Exhaustive Self-Exemplars Mining. Proceedings of the IEEE/CVF Conference on Computer Vision and Pattern Recognition.

[8] Lim B., Son S., Kim H., Nah S., Lee K.M. Enhanced Deep Residual Networks for Single Image Super-Resolution. Proceedings of the IEEE Conference on Computer Vision and Pattern Recognition Workshops.

[9] Cao Y., Wang C., Song C., Tang Y., Li H. Real-Time Super-Resolution System of 4K-Video Based on Deep Learning. Proceedings of the 2021 IEEE 32nd International Conference on Application-Specific Systems, Architectures and Processors.

[10] Cao Y., Wang C., Tang Y. Explore Efficient LUT-based Architecture for Quantized Convolutional Neural Networks on FPGA. Proceedings of the 2020 IEEE 28th Annual International Symposium on Field-Programmable Custom Computing Machines.

[11] Gomez-Rodriguez J.R., Sandoval-Arechiga R., Ibarra-Delgado S., Rodriguez-Abdala V.I., Vazquez-Avila J.L., Parra-Michel R. (2021). A Survey of Software-Defined Networks-on-Chip: Motivations, Challenges and Opportunities. Micromachines.

[12] Demirbas D., Akturk I., Ozturk O., Güdükbay U. (2018). Application-Specific Heterogeneous Network-on-Chip Design. Comput. J..

[13] Pharr M., Fernando R. (2005). GPU Gems 2: Programming Techniques for High-Performance Graphics and General-Purpose Computation.

[14] Zeng H., Cai J., Li L., Cao Z., Zhang L. (2022). Learning image-adaptive 3d lookup tables for high performance photo enhancement in real-time. IEEE Trans. Pattern Anal. Mach. Intell..

[15] Wang B., Lu C., Yan D., Zhao Y. (2021). Learning Pixel-Adaptive Weights for Portrait Photo Retouching. arXiv.

[16] Wang T., Li Y., Peng J., Ma Y., Wang X., Song F., Yan Y. Real-Time Image Enhancer via Learnable Spatial-Aware 3D Lookup Tables. Proceedings of the International Conference on Computer Vision.

[17] Jo Y., Kim S.J. Practical single-image super-resolution using look-up table. Proceedings of the IEEE Conference on Computer Vision and Pattern Recognition.

[18] Zhang Y., Li K., Li K., Wang L., Zhong B., Fu Y. Image Super-Resolution Using Very Deep Residual Channel Attention Networks. Proceedings of the European Conference on Computer Vision.

[19] Kasson J.M., Nin S.I., Plouffe W., Hafner J.L. (1995). Performing Color Space Conversions with Three-Dimensional Linear Interpolation. J. Electron. Imaging.

[20] Zhang S., Chu Y. (2009). GPU High Performance Computing: CUDA.

[21] Kirk D.B., Hwu W.M.W. (2010). Programming Massively Parallel Processors.

[22] Lu F.S., Song J.Q., Yin F.K., Zhang L.L. (2011). Survey of CPU/GPU Synergetic Parallel Computing. Comput. Sci..

[23] Huang Y., Wang Q.B., Feng J.K., Xing Z.B., Fan D., Tan X.L., Lü M.H. (2020). Rapid calculation of local topographic correction based on GPU parallel prism method. J. Surv. Mapp..

[24] Fan Z. (2020). CUDA Programming Fundamentals and Practice.

[25] Fang L., Wang M., Li D., Pan J. (2014). A CPU/GPU modulation transfer compensation method for high-resolution satellite imagery with load distribution. J. Surv. Mapp..

[26] Martin D., Fowlkes C., Tal D., Malik J. (2001). A database of human segmented natural images and its application to evaluating segmentation algorithms and measuring ecological statistics. Proceedings of the IEEE International Conference on Computer Vision.

[27] Huang J.-B., Singh A., Ahuja N. Single image super-resolution from transformed self-exemplars. Proceedings of the IEEE Conference on Computer Vision and Pattern Recognition.

[28] Wang Z., Bovik A.C., Sheikh H.R., Simoncelli E.P. (2004). Image quality assessment: From error visibility to structural similarity. IEEE Trans. Image Process..

[29] Chang H., Yeung D.-Y., Xiong Y. Super-resolution through neighbor embedding. Proceedings of the IEEE Conference on Computer Vision and Pattern Recognition.

[30] Zeyde R., Elad M., Protter M. On Single Image Scale-Up Using Sparse-Representations. Proceedings of the Curves and Surfaces–7th International Conference.

[31] Timofte R., De Smet V., Van Gool L. Anchored neighborhood regression for fast example-based super-resolution. Proceedings of the IEEE International Conference on Computer Vision.

[32] Timofte R., De Smet V., Van Gool L. A+: Adjusted anchored neighborhood regression for fast super-resolution. Proceedings of the Asian Conference on Computer Vision.

[33] Ahn N., Kang B., Sohn K.-A. Fast, accurate, and lightweight super-resolution with cascading residual network. Proceedings of the European Conference on Computer Vision.

